# Assessing harmful effects in systematic Reviews

**DOI:** 10.1186/1471-2288-4-19

**Published:** 2004-07-19

**Authors:** Heather M McIntosh, Nerys F Woolacott, Anne-Marie Bagnall

**Affiliations:** 1 Centre for Reviews and Dissemination, University of York, York, UK

## Abstract

**Background:**

Balanced decisions about health care interventions require reliable evidence on harms as well as benefits. Most systematic reviews focus on efficacy and randomised trials, for which the methodology is well established. Methods to systematically review harmful effects are less well developed and there are few sources of guidance for researchers. We present our own recent experience of conducting systematic reviews of harmful effects and make suggestions for future practice and further research.

**Methods:**

We described and compared the methods used in three systematic reviews. Our evaluation focused on the review question, study designs and quality assessment.

**Results:**

One review question focused on providing information on specific harmful effects to furnish an economic model, the other two addressed much broader questions. All three reviews included randomised and observational data, although each defined the inclusion criteria differently. Standard methods were used to assess study quality. Various practical problems were encountered in applying the study design inclusion criteria and assessing quality, mainly because of poor study design, inadequate reporting and the limitations of existing tools. All three reviews generated a large volume of work that did not yield much useful information for health care decision makers. The key areas for improvement we identified were focusing the review question and developing methods for quality assessment of studies of harmful effects.

**Conclusions:**

Systematic reviews of harmful effects are more likely to yield information pertinent to clinical decision-making if they address a focused question. This will enable clear decisions to be made about the type of research to include in the review. The methodology for assessing the quality of harmful effects data in systematic reviews requires further development.

## Background

Systematic reviews are important tools for evidence-based health care. They are certainly one of the reasons for the progress that has been made in obtaining reliable evidence on the beneficial effects of interventions. A recent study of the medical literature, using Medline and the Cochrane Library, showed that the number of systematic reviews published has increased dramatically, from a single publication in the years 1966 to 1970, to 23 in 1981 to 1985, and 2467 in 1996 to 2000 [1]. Most of the systematic reviews focused on efficacy or effectiveness. However, to make a balanced decision about any intervention it is essential to have reliable evidence on the harms as well as the benefits. Although the coverage of harmful effects has increased over time, only 27% of the reviews published between 1996 and 2000 included any information about safety, and only 4% focused primarily on the safety of the intervention reviewed [1]. This is perhaps unsurprising as many authors of systematic reviews restrict inclusion to randomised controlled trials (RCTs) to minimise bias, and harmful effects are often inadequately assessed and/or reported in RCTs [2,3]. Another important reason for the relative lack of reliable evidence on harmful effects is that RCTs are not always suitable to evaluate them and other types of study design need to be considered [4].

The methodology for conducting systematic reviews of beneficial effects from RCTs is well established, whereas the methods for systematically reviewing randomised or observational data on harmful effects are less well developed and less often used. Only 1.25% of 3604 publications cited in the 2001 edition of Side Effects of Drugs (SEDA-24) were systematic reviews [5]. At present researchers, like us, who conduct systematic reviews have limited sources of guidance, such as the suggestions offered by the Cochrane Collaboration [6]. Fortunately, research into the methodology of incorporating harmful effects data in systematic reviews is on the increase, from which we expect more sources of guidance to emerge.

It is not uncommon, even among experienced reviewers, to assume that the objective of a systematic review of harmful effects should encompass all known and previously unrecognised harmful effects and that data from all types of study design should be sought. We have re-visited three systematic reviews of drug interventions in which we had reviewed harmful effects, to evaluate our own recent experience, identify areas for improvement and to share our ideas with other researchers undertaking reviews.

## Methods

We used three reviews for this study on the basis that they had been completed recently (between 2001 and 2003) and that one of us had been the lead reviewer of harmful effects in each review. The reviews were conducted as Health Technology Assessments for the National Coordinating Centre for Health Technology Assessment (NCCHTA) on behalf of the National Institute for Clinical Excellence (NICE). The reviews, in order of completion, were: nicotine replacement therapy (NRT) and bupropion sustained release (SR) for aiding smoking cessation [7], atypical antipsychotics for schizophrenia [8], and newer antiepileptic drugs for epilepsy in adults [9].

We described and compared the methods used in each review and the problems we encountered in applying those methods. We focused our evaluation on the review objectives, the inclusion criteria for study design and the quality assessment of the primary studies. We do not report on the matter of searching for studies about harmful effects which presents another challenge to those who conduct systematic reviews [10,11], because exploratory work following from the reviews described here is underway and preliminary results are reported elsewhere [12,13].

## Results

The main components of the three systematic reviews of harmful effects are described in Table 1. Our evaluation highlighted the following aspects of the methodology that could have been improved on and others that require further development.

**Table 1:** Description of the assessment of harmful effects in the three systematic reviews

**Review**	**Schizophrenia**	**Smoking cessation**	**Epilepsy**
**Intervention evaluated**	8 atypical antipsychotics.	NRT and bupropion SR.	7 newer antiepileptic drugs.
**Objective / Scope**	Review commissioned by the HTA programme and an update of the HTA report commissioned by NICE. The objective regarding harmful effects was to determine the incidence of specific rare adverse events to populate an economic model.	Scope provided by NICE: To review all known or unknown harmful effects that might be associated with the interventions.	Scope provided by NICE: To include adverse effects in a review of RCTs of clinical effectiveness in adults with epilepsy. The reviewers undertook a supplementary review of serious, rare and long-term harmful effects. Serious was defined by WHO criteria [25], long-term as longer than 6 months, and rare as defined by the authors of primary studies.
**Study designs included**			
***Randomised trials***	RCTs of atypical antipsychotics versus alternative drug treatment or placebo in schizophrenia.	An existing Cochrane review was used as a source of summary data on adverse effects from RCTs of effectiveness [26]. Studies that assessed safety as the primary objective were included in the review of primary studies of harmful effects. This included RCTs that investigated aspects of clinical pharmacology that might impact on the drugs' tolerability and safety.	The five most commonly reported adverse effects were extracted from RCTs as part of the review of clinical effectiveness in epilepsy. RCTs in indications other than epilepsy and dose comparisons were eligible for inclusion in the supplementary review of harmful effects.
***Non-randomised studies***	Cohort studies and case series with 2000 or more participants or at least 2 years follow-up, and case-control studies of any size or duration.	Uncontrolled trials, prospective and retrospective observational studies, data from adverse events monitoring systems (e.g. UK yellow card scheme) and case reports.	Non-randomised controlled trials, cohort and case-control studies, prospective case series and other uncontrolled trials, and open-label extension phases of trials. More than 300 participants had to be exposed or follow-up more than 6 months unless the study objective was to investigate a specific adverse effect. Prescription event monitoring [27], and post-marketing surveillance reports were also included.
**Studies identified**	6477 items screened, 924 articles retrieved, and 223 studies included: 171 RCTs, 13 cohort studies, 1 case-control study, 38 case series.	1280 items screened, 353 articles retrieved, and 123 studies included: 25 RCTs, 4 non-randomised controlled trials, 30 uncontrolled trials, before/after studies or cohort studies, 1 case-control study, 9 surveillance studies, 1 survey, 53 case reports or case series.	108 RCTs were included in the review of effectiveness, selected from 4211 items screened and 887 articles retrieved. In the supplementary review of harmful effects 3884 items were screened, 227 articles retrieved, and 77 studies included: 2 RCTs, 2 non-randomised controlled trials, 26 uncontrolled trials, 14 open-label phases, 25 cohort studies, 1 case-control study, 4 prescription event monitoring studies, 3 post-marketing surveillance studies.
**Quality assessment**	Quality checklists for various study designs provided in CRD Report 4 were used [28].	The quality checklist for RCTs provided in CRD Report 4 [28], and checklists for the other study designs published elsewhere [29], were used.	Published checklists were used as a starting point. Questions were amended and others added to capture information specifically on the reliability of harmful effects data.
**Findings of review**	Very few studies with useful data were found, so the economic model could not be populated with incidence rates of the adverse events of interest.	Primarily the findings merely reflected the accepted side-effect profiles for NRT and bupropion SR. The review did not identify any previously unknown harmful effects.	The supplementary review of harmful effects did identify reports of potential adverse effects not reported in the RCTs of clinical effectiveness. However, these were mostly effects already documented in tertiary sources. There was insufficient evidence to attribute causality of other reported effects to the test drugs.

### Review objectives

The schizophrenia review objective appeared to be appropriate in seeking to determine the incidence of named outcomes that were considered by health economists to be most likely to lead to a change in prescribed treatment [14]. The objectives of the smoking cessation and epilepsy reviews were very broad in comparison. Given that the side-effect profiles of the drugs for smoking cessation were well established, with details available in various published standard reference texts [15,16], it would have been more efficient to focus the review effort on a clear question, such as the significance of seizures for bupropion SR and the cardiovascular effects of nicotine in NRT. The objective of the review of harmful effects of the antiepileptic drugs did not target clinical decision-making; the supplementary review of harmful effects might have been of real use to decision makers if we had focused on a crucial clinical question such as the safety of the drugs in pregnancy.

### Study designs

All three reviews included study designs other than RCTs to assess harmful effects. The types of non-randomised studies included for each review reflected differences in the reviews' objectives, our judgment as reviewers as to where the most useful data were likely to be found, and was to some extent pragmatic in terms of the time available to complete the reviews. The reviews with the broad objectives included more non-randomised studies and more diverse study designs. The schizophrenia and epilepsy reviews specified a minimum size and duration of study to be included (see table) in an attempt to add data over and above what was available from the largest and longest RCTs. Doing this did involve some indeterminable risk of missing important information.

The review of observational studies carried out in the schizophrenia review was necessary because the pre-determined harmful effects of interest were known to be under-reported in RCTs [8]. The inclusion of non-randomised studies in the smoking cessation review might have targeted observational data on specific questions about harmful effects had we reviewed beforehand the RCTs that were summarised briefly in the Cochrane review. Similarly in the epilepsy review all the adverse events (not just the most common) reported in the RCTs of clinical effectiveness should have been reviewed before moving on to observational studies.

### Applying the inclusion criteria

Once the inclusion criteria for study design had been defined, applying them was problematic. Reports of primary studies rarely described the study design in sufficient detail. Many of the studies included in the schizophrenia review purported to be cohort studies but on closer examination were in fact large case series involving more than one intervention. Some of the 'cohort study' data on bupropion SR included in the smoking cessation review had actually been derived retrospectively from RCTs. How exactly the 'cohorts' had been established in studies of epilepsy was often unclear in terms of the source population, eligibility criteria, and selection, or was simply not reported. Had we, in all three reviews, only included reports of studies fitting textbook definitions of particular study designs, virtually all of the primary study reports we identified would have been excluded. The inclusive approach we took turned out to be unrewarding.

In the smoking cessation review, in addition to difficulties with the study design inclusion criteria, application of the criterion to only include studies in which assessment of adverse events was the primary objective was problematic because it involved a high degree of subjective judgment.

### Quality assessment

We encountered problems when applying published checklists in our reviews of harmful effects. The response to some questions depended on the outcome of interest, for example, follow-up may have been adequate for the assessment of the primary (usually a beneficial) outcome of the study but not for the collection of data on harmful effects. We also found that published checklists omit key features such as how harmful effects data were recorded. In the epilepsy review we were in a position to learn from the earlier reviews and spent time clarifying the questions in the checklists so that they would provide information relevant to the reliability of the harmful effects data. We also added items pertinent to reports of harmful effects such as how and when events were recorded and whether the time at which they occurred during the study was reported. Although this informed approach was a step in the right direction, the major hindrance to applying checklists in all three reviews was inadequate reporting of the basic design features of the primary studies.

Once the quality criteria had been applied there remained the challenge of interpreting the results. In our reviews we described the evidence identified and tabulated the response to each checklist question for each primary study. This generated lengthy summaries that had limited utility. Even comparing validity within study designs (not across them) we found it impossible to synthesise the information as all the included studies had methodological flaws and features that could not be assessed due to inadequate reporting. Reaching a decision about which studies were likely to give the most reliable results was not straightforward.

## Discussion

Our experience of reviewing harmful effects mirrors that of other researchers in that a significant investment of effort failed to yield significant new information [6,17].

A focused review question is standard practice for assessing beneficial outcomes in systematic reviews and should also be so when reviewing harms. Researchers conducting reviews need to make sure that they address a well-formulated question about harms that are likely to impact on clinical decisions. Focusing a review question about harmful effects will not necessarily mean restricting it to specific adverse events but may mean, for example, addressing a particular issue such as long-term effects, drug interactions, or the incidence of mild effects of importance to patients. If the aim of the research is to look for previously unrecognised harmful effects, analysis of primary surveillance data may be more appropriate than a systematic review [18]. Researchers also need to be aware that scopes set by external commissioning bodies, despite having consulted with national professional and patient organisations, may not be a suitable question to address in a systematic review. The wisdom of broad and non-specific questions about harmful effects should be questioned because the resources, especially time, needed to do this comprehensively are usually insufficient.

It is important to realise that an unquestioning belief that observational studies are the best source of harmful effects data simply because they are not RCTs can be a pitfall. It is essential to think carefully about the review question before widening the inclusion criteria to include non-randomised study designs. Some harmful effects, such as very rare events or those emerging in the long-term, are unlikely to be addressed adequately in RCTs. But, even if observational studies are appropriate to the review question researchers should be prepared for the difficulty of interpreting observational study data outweighing the anticipated benefits.

The importance of quality assessment of RCTs in systematic reviews of effectiveness is well established [19], but debate continues over the usefulness of checklists and scales. Quality assessment of other study designs in systematic reviews is far less well developed [20]. Although the feasibility of creating one quality checklist to apply to various study designs has been explored [21], and research has gone into developing an instrument to measure the methodological quality of observational studies [22], and a scale to assess the quality of observational studies in meta-analyses [23], there is as yet no consensus on how to synthesise information about quality from a range of study designs within a systematic review. Our appraisal of our reviews has shown that these difficulties are compounded when reviewing data on harms.

It is essential that quality assessment is able to discriminate poor from better quality studies of harmful effects. Levels of evidence hierarchies have several shortcomings. The hierarchy of evidence is not always the same for all harmful or beneficial outcomes. For example, an RCT with adequate internal validity but limited sample size or follow-up may be a less reliable source of information about relatively uncommon harmful effects emerging in the long-term than a large well-conducted cohort study with many years of follow-up. Another problem with ranking evidence in a hierarchy is that different dimensions of quality get condensed into a single grade, resulting in a loss of information. Furthermore, the dimensions included in current hierarchies may not be the most important in terms of reflecting the reliability of a particular study's findings [24]. Researchers need to clarify *a priori *what exactly they need to glean from their quality assessment of the primary studies in their own review of harmful effects and it may be necessary to differentiate clearly between internal and external validity.

We suggest that further research is needed to collate, assimilate and build on the existing information relevant to systematically reviewing primary studies for harmful effects of health care interventions. This should include a review of the literature pertinent to the methodology of incorporating evidence of harmful effects in systematic reviews; a description and categorisation of the methods used in systematic reviews published to date, and any evidence from methodological research on which they are based; and the development of quality assessment methods.

## Conclusions

Appraisal of our recent experience highlighted some of the problems inherent in conducting systematic reviews of harmful effects of health care interventions. Such reviews need to address a well-formulated question to facilitate clear decisions about the type of research to include and how best to summarise it, and to avoid repeating what is already known. The review question about harmful effects needs to be relevant to clinical decision-making. A systematic review of the methodology pertinent to systematic reviews of harmful effects is warranted.

## Competing interests

None declared.

## Authors' contributions

AMB, HMM and NFW conducted the review work described. NFW conceived of the study. NFW and HMM drafted the manuscript. All authors contributed to, read and approved the final manuscript.

## Pre-publication history

The pre-publication history for this paper can be accessed here:


